# A gene-signature progression approach to identifying candidate small-molecule cancer therapeutics with connectivity mapping

**DOI:** 10.1186/s12859-016-1066-x

**Published:** 2016-05-11

**Authors:** Qing Wen, Chang-Sik Kim, Peter W. Hamilton, Shu-Dong Zhang

**Affiliations:** Centre for Cancer Research and Cell Biology (CCRCB), Queen’s University Belfast, Belfast, UK; Scientific Computing Department, STFC Daresbury Laboratory, Daresbury, Warrington, UK; Northern Ireland Centre for Stratified Medicine, University of Ulster, C-TRIC Building, Altnagelvin Hospital campus, Glenshane Road, BT47 6SB, Derry/Londonderry, UK

**Keywords:** Connectivity mapping, Differentially expressed genes, Gene signature progression, Disease inhibitory compounds, Lung cancer, Breast cancer

## Abstract

**Background:**

Gene expression connectivity mapping has gained much popularity recently with a number of successful applications in biomedical research testifying its utility and promise. Previously methodological research in connectivity mapping mainly focused on two of the key components in the framework, namely, the reference gene expression profiles and the connectivity mapping algorithms. The other key component in this framework, the query gene signature, has been left to users to construct without much consensus on how this should be done, albeit it has been an issue most relevant to end users. As a key input to the connectivity mapping process, gene signature is crucially important in returning biologically meaningful and relevant results. This paper intends to formulate a standardized procedure for constructing high quality gene signatures from a user’s perspective.

**Results:**

We describe a two-stage process for making quality gene signatures using gene expression data as initial inputs. First, a differential gene expression analysis comparing two distinct biological states; only the genes that have passed stringent statistical criteria are considered in the second stage of the process, which involves ranking genes based on statistical as well as biological significance. We introduce a “gene signature progression” method as a standard procedure in connectivity mapping. Starting from the highest ranked gene, we progressively determine the minimum length of the gene signature that allows connections to the reference profiles (drugs) being established with a preset target false discovery rate. We use a lung cancer dataset and a breast cancer dataset as two case studies to demonstrate how this standardized procedure works, and we show that highly relevant and interesting biological connections are returned. Of particular note is gefitinib, identified as among the candidate therapeutics in our lung cancer case study. Our gene signature was based on gene expression data from Taiwan female non-smoker lung cancer patients, while there is evidence from independent studies that gefitinib is highly effective in treating women, non-smoker or former light smoker, advanced non-small cell lung cancer patients of Asian origin.

**Conclusions:**

In summary, we introduced a gene signature progression method into connectivity mapping, which enables a standardized procedure for constructing high quality gene signatures. This progression method is particularly useful when the number of differentially expressed genes identified is large, and when there is a need to prioritize them to be included in the query signature. The results from two case studies demonstrate that the approach we have developed is capable of obtaining pertinent candidate drugs with high precision.

**Electronic supplementary material:**

The online version of this article (doi:10.1186/s12859-016-1066-x) contains supplementary material, which is available to authorized users.

## Background

Over the past few years gene expression connectivity mapping has gained much popularity among biomedical researchers because of its promising applications as demonstrated by an increasing number of studies in different research areas: drug discovery [1–5], drug re-positioning [6–8], predictive toxicology [9], and chemical carcinogenicity assessment [10], to name a few. The Connectivity Map (CMap) concept was first introduced by Lamb et al. [11] with the idea of using gene expression profiles/signatures to represent different biological states, and then to establish the connections among these states based on their gene expression patterns. The establishment of a connection between two biological states may have different implications. If the connection was made because the key set of genes were similarly up- or down-regulated in the two states, it may indicate that the two states have the same activated biological processes or pathways, such as the autophagy process and an autophagy enhancing compound fasudil identified in [12], and consequently such a connection would be very useful for gaining insights into the underlying mechanism of one state based on the knowledge of the other, and vice versa. On the other hand if the connection was made because the key set of genes were oppositely regulated, it may indicate that the two states negate each other. If one is an undesirable state such as a disease, and the other is a chemical compound-induced state, the compound may be useful to treat that particular disease [13], as in the case of application to inflammatory bowel disease [14], and human skeletal muscle atrophy [15]. From a user point of view, one important question often asked is: what is the best way of making a gene expression signature that can represent the biological state of interest accurately and be able to return meaningful biological connections?

Within the gene expression connectivity mapping framework, there are three key components: reference gene expression profiles, query gene signatures, and expression pattern matching algorithms. To date, much of the methodological development has been focused on the first and the third components above [16–20]. One important aspect related to the development has been the efforts to reduce the high number of possible confounding factors and consequent batch effects within the heterogeneous CMap datasets, eg., by utilising filtering and normalisation steps to improve the signal to noise ratio [19], or by explicitly considering the variability in the transcriptional response and capturing the “consensus” response to a compound across multiple cell lines and dosages [20]. In this paper, we are mainly concerned with the construction of query gene signatures, which was previously left to users without much guidance. For discussing the rationale of the “gene signature progression” method to be introduced, we first outline the differences between reference profiles and gene signatures in the context of connectivity mapping.

The idea of using some form of molecular profile to characterize a biological state is not completely new. In essence this idea was the basis of many clinical practices. For example, various biochemical tests measure the molecular profile of a patient, which can then be used to infer or learn about the biological state of the patient (being with a particular disease or healthy). The main assumption behind the connectivity mapping concept is the generalization of molecular profile (bio-marker) measurement. In this case the bio-marker molecules are the messenger RNAs of genes in an organism. Because of the sheer number of different mRNAs measured, they collectively provide a much richer and complex description of the biological state than a few marker molecules could achieve. Theoretically if we have identified all the different molecules and their abundance in a cell, we will have an exact specification of the cell’s biological state. The main assumption underlying the connectivity mapping concept was that the specification of the mRNA profiles as measured by microarrays can provide an adequate description of the biological state. With this premise, we discuss here the roles played by reference profiles and gene signatures in the connectivity mapping framework.

As described previously [11, 16], a reference gene expression profile contains the full list of genes that were measured by the microarrays, and specifies their abundance in one condition relative to another. Because a reference profile contains information about each gene, it is intended to provide a comprehensive description of the biological state it represents. A query gene signature, on the other hand, is a short list of genes that capture the most important features of a biological state. Its difference to a reference profile is that a gene signature is not meant to provide the full description of how each gene has changed its expression, but rather to provide the most prominent features in that biological state. Obviously any biological condition (state) will have many features/aspects, but they are not of equal importance. For establishing the connection between two biological states, one needs to decide what features to include in the gene signature. The aim of gene signature construction is to compile a list of genes that collectively capture the most prominent features of the biological state it is intended to represent. Here in this paper we propose a two-stage process to achieve that goal.

The initial input is assumed to be from microarray gene expression data that have been properly processed through some common steps, such as quality control, background correction, normalization, scaling or transformations, where appropriate. These pre-processing steps are very important in microarray data analysis and some recent research focused on these aspects, eg [21, 22], but they are not in the scope of the present work. Our starting point is that we have obtained the gene expression dataset, from which the expression levels of the genes can be quantified in the available biological samples. Given these data, we propose the following two-stage process for the construction of gene expression signature in connectivity mapping: (1) differential gene expression analysis to provide an ordered list of significant genes. (2) gene signature progression analysis to systematically determine the minimum length of the gene signatures able to return significant connections to reference drugs with an acceptable false discovery rate. Detailed description of the approach is presented in the following “Methods: a two-stage process” section.

## Methods: a two-stage process

**Fig. 1:**
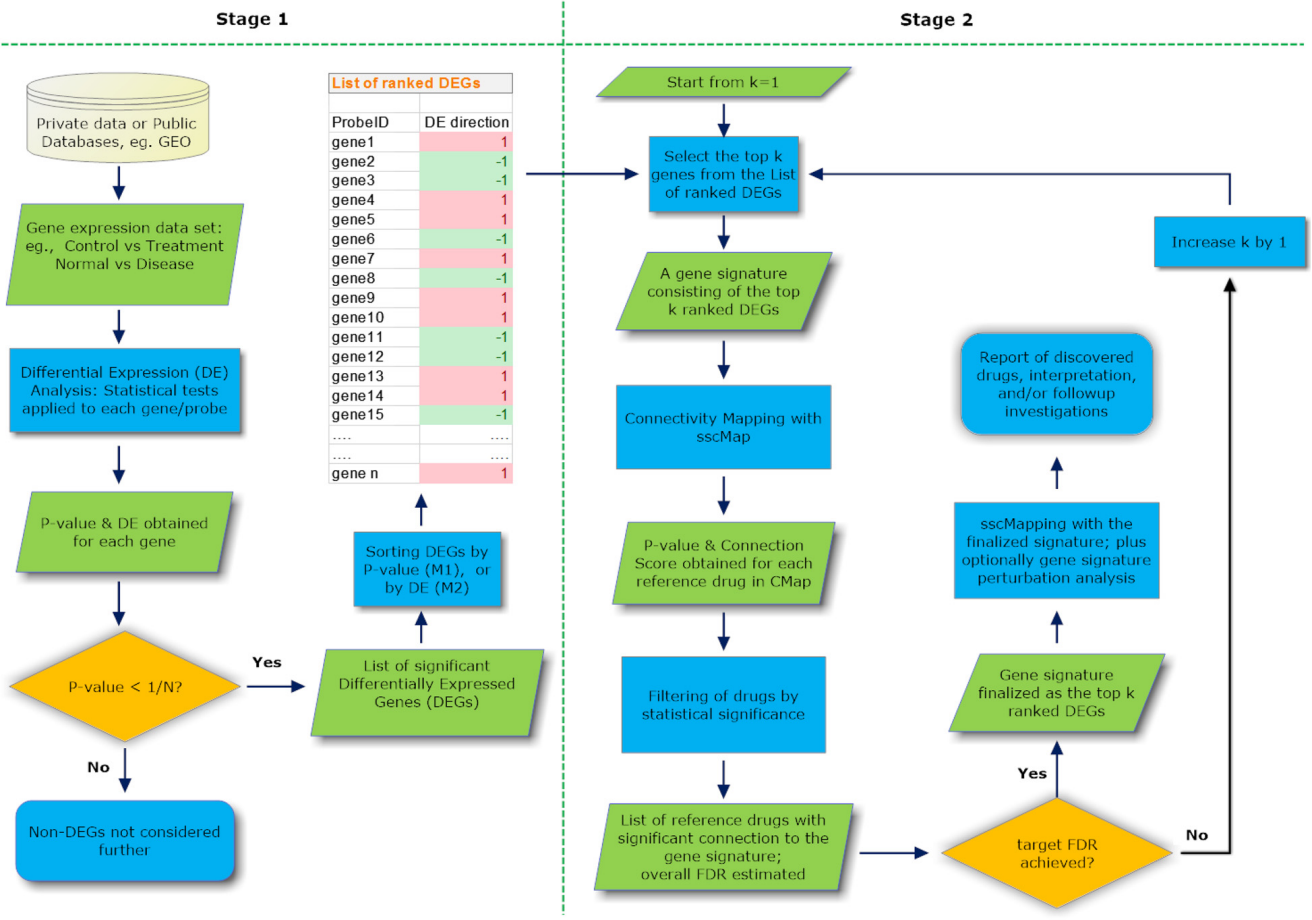
Flow chart of the two stage process. The output from stage 1 is a ranked list of significant genes with their regulation status (direction of differential expression in the treatment condition relative to the control). This is used as input to stage 2, gene signature progression. Starting from *k*=1, the top *k* genes are fed to sscMap as a query signature to pull out significant drugs. This process is run iteratively with increasing *k* until a pre-set target FDR is achieved for the returned significant drugs

A schematic workflow of the two-stage process is depicted in Fig. 1. The aim of the first stage, differential expression analysis, is to derive a ranked list of DEGs (Differentially Expressed Genes) together with their regulation status (direction of differential expression in the treatment condition relative to the control), which can serve as an input to the general connectivity mapping process. The second stage, gene signature progression analysis, involves creating multiple gene signatures consisting of an increasing number of top ranked genes, and then querying the sscMap iteratively with gene signatures of increasing length until a number of significant drugs are returned with a pre-set acceptable false discovery rate.

The sscMap connectivity mapping framework was developed previously to introduce a more principled statistical test in connectivity mapping [16, 17]. It was bundled with 6100 compound-induced reference gene expression profiles as its core database. When a user-supplied query gene signature is presented to it, sscMap calculates a connection score between the query signature and each set of reference profiles in the core database, then performs computationally intensive permutation tests, to assess the statistical significance of each observed connection score. A number of drugs with significant connection to the query signature are then returned as the results of this process.

### Differential gene expression analysis

In general, differential gene expression analysis involves two or more biological conditions. For gene signature construction in connectivity mapping, we are mainly concerned with cases where they are two conditions. One of them is a control condition which serves as a reference point. The other condition is the state of our interest, for example, a disease state or a state as a result of some form of biological, chemical, or genomic perturbation experiment. This is similar to the construction of reference gene expression profiles, where a vehicle control condition and a drug treated condition are required.

An important issue in the differential gene expression analysis is the multiple testing correction that must be considered when conducting a large number of statistical tests at the same time. When tens of thousands of genes are being tested in the same analysis, the conventional statistical significance level of 0.05, which was developed for single statistical test, is no longer adequate. Purely by chance, 5 *%* of the genes tested will turn out to have a *p*-value less than 0.05 even if there is no difference at all between the two biological conditions being compared. To safeguard the findings of differential gene expression against too many false positives creeping in, the false discovery rate (FDR) has become a commonly accepted measure to control in situations where multiple hypothesis testing is involved [23]. This measure allows users to estimate the number of false positives given the total number of positive results obtained. An equivalent way of achieving this goal is to control the expected number of false positives directly, and then calculate the empirical false discovery rate. For example, to control the expected number of false positives to be *E*_*fp*_, one can set the threshold *p*-value as *E*_*fp*_/*N*, where *N* is the total number of hypotheses being simultaneously tested, and in the case of microarray differential gene expression analysis, *N* is the total number of genes (probesets) measured by the microarrays. In this paper we set *E*_*fp*_=1 to apply a tight control on the expected number of false positives in differential expression analysis. This is very important because it ensures we can have the confidence in the differentially expressed genes declared.

With differential expression analysis providing a list of significant genes, here comes a key guiding principle of gene signature construction: **only genes that are differentially expressed with stringent statistical significance should be passed on to the next stage.** One immediate question is: what if the number of significant genes in the differential expression analysis is so small that no significant connections could be established in the subsequent connectivity mapping step. In such a situation, the guiding principle above should still prevail. A more frequently encountered situation nowadays is that the microarray experiments included a large number samples and the studies were adequately powered, and a long list of genes have passed the significance criteria in the differential expression analysis. But should we include all of them in the subsequent connectivity mapping step? Is it necessarily the better if we include more genes in the query signature? To address these issues, the second step “gene signature progression” comes into play.

### Gene signature progression

Given a long list of differentially expressed genes (DEGs) obtained rigorously, how should we proceed to gene expression connectivity mapping using these genes as input? Before describing the detailed procedure of gene signature progression, it is useful to outline the rationales and motivation behind the idea. The gene signature progression procedure was conceived based on the following argument: although a long list of significant genes have been identified with high statistical rigor, these genes are not equally important in characterizing the biological state of interest, and hence they should have different weights. We need a way to quantify the importance and contribution of these genes. Here we adopt similar guiding principles used in the construction of reference gene expression profiles for sscMap. Briefly, (1) up-regulated genes and down-regulated genes should be treated on a equal-footing basis. (2) genes that are differentially expressed to a greater extent or those with greater significance should have more weight and thus receive higher ranks. Note that in Reference [16] genes were sorted by the absolute value of their log expression ratio (drug-treated state over vehicle control). In the present case of gene signature construction we have the option of using statistical significance *p* value to sort and rank the genes. Here we describe two ways of sorting the genes and ranking them.

- M1: sorting the genes by their *p* values
  The first natural solution to ranking the genes is to sort them by their *p* values in ascending order, with the smallest *p* value ranked the highest. The rationale behind this method is that the smaller the *p* value, the more significant the differential gene expression.
- M2: sorting the genes by their absolute log expression ratios
  The argument supporting this method is that all these genes have passed the statistical significance criteria in the differential expression analysis step, and they are all genuine differentially expressed genes. Now it is a matter of ranking them based on their biological effects. In this case, the absolute value of log expression ratio is chosen as the closest proxy to quantify their biological effect.

It should be noted that the criteria in M1 and M2 are not independent of each other, but they are strongly correlated. In achieving a small *p* value in the differential gene expression analysis, the magnitude of differential expression (the log ratio) must be reasonably large as compared to its variance. Large log ratio or small variance alone would not necessarily lead to statistical significance. One could argue that Method M1 is already well balanced because the *p* value already contains information on the magnitude of differential expression and the consistency of the gene’s behavior across replicates. In the two case studies of this paper, we applied M1 in sorting and ranking the genes.

After the genes have received their absolute ranks using one of the methods above, they are given signed ranks which are just the absolute ranks multiplied by +1 or −1 depending on the direction of their differential expression, in a similar way as in the construction of reference gene expression profiles [16]. Once signed ranks have been assigned to all genes in the DEG list, we use this gene list as an input to subsequent connectivity mapping analysis, assuming that the genes with the highest (absolute) ranks (regardless of signs) represent the most important features of the biological state.

In order to return the most relevant connections to our biological state of interest, we devised a “gene signature progression” procedure to systematically determine the minimal (optimal) length of the gene signature that can accurately represent the biological state. The gene signature progression method proceeds as follows:

- Forming a gene signature
  Given a ranked list of genes, we make a gene signature consisting of the top *k* ranked genes, denoted by **s**_*k*_, where *k* is an integer from 1 to *n*, and *n* is the total number of genes in the initial DEG list passed on from the differential gene expression analysis.
- Querying sscMap
  We use the signature **s**_*k*_ as an input to query the sscMap, which returns the connection scores of **s**_*k*_ to all the reference profile sets (drugs) and their corresponding *p*-values.
- Calculating the empirical FDR of the returned connections
  We set a threshold *p*-value to determine if any returned connections are statistically significant. In our analysis the threshold *p*-value is set as *N*_*fp*_/*N*_*sets*_, where *N*_*fp*_ is the number of false connections we are prepared to tolerate and expected to have in the results, and *N*_*sets*_ is the total number of reference sets we are querying. Under this setting if we observe *N*_*p*_ as the total number of significant connections, we use *N*_*fp*_/*N*_*p*_ to estimate the empirical false discovery rate (eFDR) [24]. In this way for each run of connectivity mapping using a gene signature **s**_*k*_, we get an eFDR, which is a function of the signature length *k*.
- Progressively looping over the gene signatures **s**_*k*_’s
  Starting from *k*=1 with step increment until *k*=*n*, to obtain the eFDR for each **s**_*k*_; break out from the loop when the first eFDR ≤*α* is observed, where *α* is a pre-set FDR threshold.
- Target FDR achieved
  Take the break-out *k* from the previous step as the optimal length of the gene signature.

It is possible that after looping over all the progression signatures, none has returned an eFDR ≤*α*. In such cases, the gene signature with the smallest eFDR could be taken as the optimal one. This is not ideal in terms of the eFDR achievable, but nevertheless the result is just a reflection of the underlying biology. From a user’s point of view, one has then to decide, given the achieved false discovery rate, whether one wants to follow up the findings or not.

## Results

### A “simulated experiment”

First, we carried out a “simulated experiment” to see if the gene signature progression approach indeed allows the capture of the most prominent feature of the biological state for which we know the truth. For this purpose, we created a gene signature for histone deacetylase (HDAC) inhibitor based on the internal reference profiles of CMap database. Among the over 1300 drugs profiled in the camp reference dataset, the following drugs are known to be HDAC inhibitors (HDACi): vorinostat, trichostatin A, valproic acid, HC toxin, sodium phenylbutyrate, scriptaid, and MS-275. The number of individual reference profiles for these drugs are listed in Table 1. To obtain an adequate sample size and also to avoid possible dominance of results by the drugs with large number of replicates, eg, trichostatin A and valproic acid, we used the following four drugs: HC toxin, sodium phenylbutyrate, scriptaid, and MS-275, which have relatively small numbers of replicates in the dataset, to create the HDAC inhibitor gene signature. We combined their data to obtain a single list of ranked significant genes that are associated with these HDAC inhibitors (see Additional file 1 for the full list of 104 significant genes and their rank order). The genes were then sorted by the absolute values of their sum rank scores, and finally we obtained an ordered list of genes with signed ranks. As this was the result of combing four HDAC inhibitors specified above, this gene list was expected to represent the biological state(s) as induced by these HDAC inhibitors. Using this list of ranked genes as input, we performed the gene signature progression analysis, *k*=5 was determined to be the minimum length of gene signature that returned a number of significant drugs connection with an overall FDR no greater than 0.1. The results of sscMap connectivity mapping analysis using this minimum length gene signature are presented in Table 2. As can been seen from this table, not only the four input drugs, HC toxin, sodium phenylbutyrate, scriptaid, and MS-275, were identified as significantly connected to the gene signature (as they were expected to be), the other major HDAC inhibitors, vorinostat, trichostatin A, and valproic acid were also pulled out as significantly connected to the HDACi signature. It should be emphasized that the reference data for these 3 drugs, were not involved at all in creating the HDACi gene signature. So these results are reassuring because the gene expression connectivity mapping framework and the gene signature progression approach has worked very well in this “simulated experiment”. From this experiment, we see the added value of the gene signature progression approach to connectivity mapping. As designed, it helped to capture and highlight the most important feature of the biological state being studied. In this case, it was HDAC inhibition. Without the gene signature progression step, an arbitrarily chosen signature length would be often used, for example, *k*=50 or *k*=100. Using signatures of these lengths to perform gene expression connectivity mapping, these HDAC inhibitors would still be returned as significant connections, but they were returned together with many other drugs (see Additional file 2 for the results of sscMaping using the *k*=50 HDACi signature). Due to the co-existence of many other seemingly significant drugs, the main biological theme (HDAC inhibition) could potentially be weakened or even masked. Therefore, this example demonstrates that the signature progression approach does what it was designed for: to help users capture the most important biological theme with sharp focus and high precision.

**Table 1:** Known HDAC inhibitors in the CMap reference dataset

Compound	Replicates
Vorinostat	12
Trichostatin A	182
Valproic acid	57
HC toxin	1
Sodium phenylbutyrate	7
Scriptaid	3
MS-275	2

**Table 2:** The significant connections returned from sscMap using the HDACi optimal gene signature (*k*=5) obtained from the “simulated experiment”

Compound	Replicates	cscore	*p* value	zscore	SM	PS
Sodium phenylbutyrate	7	0.684	5.0E-06	7.93	1	1
Trichostatin A	182	0.822	5.0E-06	6.18	1	1
Scriptaid	3	0.928	5.0E-06	5.39	1	1
Valproic acid	57	0.323	1.0E-05	5.31	1	1
Rifabutin	3	0.880	5.0E-06	5.21	1	1
Vorinostat	12	0.890	5.0E-06	5.19	1	1
MS-275	2	0.954	5.0E-06	4.30	1	1
Withaferin A	4	0.729	2.0E-05	3.97	1	1
HC toxin	1	0.928	5.0E-06	3.59	1	1
2-deoxy-D-glucose	1	0.902	1.0E-05	3.49	1	1

All 7 known HDAC inhibitors, including those not used in signature generation, were pulled out. SM (significance mark) =1 indicates that the connection *p* value is less than the preset threshold *E*_*fp*_/*N*_*sets*_=1/1309≈7.6×10^−4^; PS (perturbation stability) is also shown here

### A case study with lung cancer

As an example application of the gene signature progression method introduced here, we analyzed a public dataset from an independent study [25], where the tumor and paired normal tissues from 60 non-smoker female lung cancer patients were profiled using Affymetrix Human Genome U133 Plus 2.0 (HG-U133_Plus_2) microarrays. We downloaded the 120 raw Affeymetrix CEL files from the Gene Expression Omnibus (GEO) dataset GSE19804. For connectivity mapping analysis we wanted to compile a gene signature describing the deviation of tumors from normals, so that we could have an accurate representation of the cancerous state. Raw microarray data were pre-processed using the Affymetrix MAS5 algorithm with the scaling parameter sc = 500 as implemented in the R package affy [26]. We then worked on the log2 scale by transforming MAS5 values to log2MAS5. As the reference profiles in the connectivity mapping database were generated using Affymetrix Human Genome U133A (HG-U133A) microarrays, the common probesets (22277 in total) between the HG-U133_Plus_2 and the HG-U11A arrays were extracted. A paired-sample t test was applied to each of the common probesets to identify which is differentially expressed between the lung cancer condition and the normal condition. With a sample size of 120 the study was well powered for identifying differential gene expression. As a result, 6313 probesets had a *p*-value less than the threshold discussed above *E*_*fp*_/*N*=1/22277≈4.5×10^−5^. This gave an estimated eFDR of 1/6313≈1.6×10^−4^, so we were very confident that these differentially expressed genes all reflected some aspects of the real biology. But feeding all these genes as part of the gene signature to connectivity mapping would be difficult as the gene signature is too long and it would overwhelm the connectivity mapping algorithm. It is also very difficult for human comprehension to include such a large number of “features” if one wants to get a better grasp of the biology under study. This example demonstrates the needs for some form of filtering and selection procedure so that the major theme(s) of the biology be captured and the big picture is not buried under many minor details. To this end we set to filter out the probesets that were less prominent. We took the following steps to shorten the list of selected genes: (1) Only genes that met the statistical significance criteria were passed on to the next step; those regarded as statistically non-significant were not considered further. In this case we set to tolerate an expected number of false positives *E*_*fp*_=1 as outlined above. (2) Following the previous step, we required genes to have a minimum mean expression level across the two conditions. Specifically, genes with mean log2MAS5 values less than 6 in both conditions were excluded from further analysis. This minimum value 6 for log2Mas5, although somewhat arbitrary, was based on our extensive experience dealing with microarray data. The rationale of this filtering was that for genes with low expression levels in both conditions, we were less confident about their differential expression status, and also because of their low expression levels, their biological significance was considered less important than those with higher expression. (3) Following the previous step, we required genes to have a minimum expression difference between the two conditions. Specifically, genes with an absolute expression difference less than 1 were excluded from further analysis. As we work on log2 scale data, an absolute difference of 1, is equivalent to a fold change (ratio) of 2 in MAS5 expression values. So essentially we required genes to have a minimum fold change of 2, either up or down.

After the three filtering steps described, we still had 1779 probesets to work with. These served as our input DEG list to the gene signature progression analysis with connectivity mapping. These 1779 probesets were sorted in ascending order of their *p*-values, the ones on top of the sorted list were regarded as most important. We were interested in compounds with potential inhibitory effects on the tumorous state, therefore we looked for the connections with negative connection scores and used a one-sided test to calculate the *p*-value associated with the signature-to-drug connection. Briefly, for an observed connection score between the query signature and a reference expression profile, we generated 10^5^ random gene signatures of the same length, and calculated the connections scores between these random signatures and the reference profile. Each random gene signature is just a list of genes randomly sampled (without replacement) from all genes profiled in the reference dataset, with their up- or down- regulation status randomly set, as detailed in [16]. The proportion of random scores that were less than the observed connection score gave an estimate of one-sided (left-sided) *p*-value, which was then used to determine whether the observed score was statistically significant or not. In our connectivity mapping analysis, the query gene signature was compared to each set of reference profiles derived from the same compound; a connection score between the query signature and each reference set was calculated and the corresponding left-sided *p*-value was estimated as described above. The results returned from sscMap with a given query signature included the list of compounds examined, their connection scores to this query gene signature, and the associated *p*-values.

**Fig. 2:**
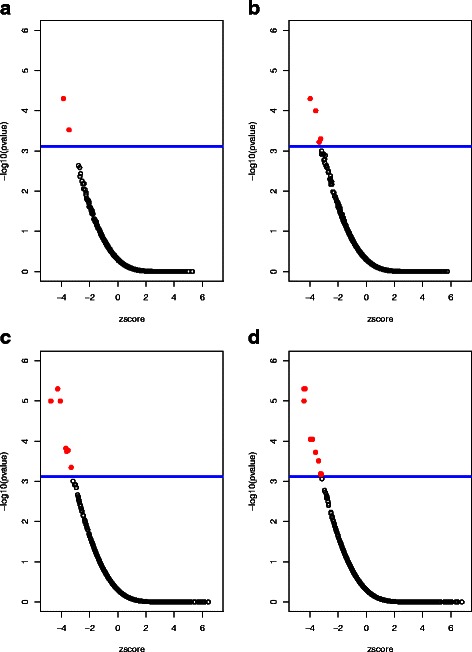
Snapshots of the gene signature progression process in the lung cancer case study. Each panel shows results of sscMap with the top *k* ranked genes as the query signature, with *k*=150,200,300, and 322, respectively for (**a**)–(**d**). This figure exemplifies the intermediate results of individual steps in the gene signature progression process. The *blue horizontal line* indicates the position corresponding to the threshold *p*-value. Any points above this line are considered statistically significant. The number of compounds significantly connected to the query gene signature (*solid red circles*) are 2,4,7, and 10 respectively for panels (**a**)–(**d**). *k*=322 is the optimal signature length at which the preset FDR target ≤0.1 was first achieved

We followed the gene signature progression procedure by generating progression signatures of increasing length *k* consisting of the top *k* probesets from the sorted DEG list obtained. Our target false discovery rate was set moderately as 0.1. Figure 2 shows a few snapshots in the gene signature progression process. Panel (C) of this figure, for example, shows the results of sscMap using the gene signature **s**_300_ as the query input, consisting of the top 300 ranked genes from the differential expression analysis. As can be seen in this case, 7 compounds were above the horizontal line, the position corresponding to the threshold *p*-value. As we expected 1 false connections among these significant compounds, the empirical FDR was calculated as 1/7≈0.14, which was higher than the pre-set FDR target 0.1. Therefore we needed to progress the gene signature further after this *k*=300 sscMap run. By following the gene signature progression procedure, we found that *k*=322 was the minimum signature length that returned significant connections with an eFDR ≤0.1. This list of gene was taken as our optimal gene signature for the lung cancer-vs-normal comparison (See Additional file 3 for the full list of these 322 genes and their ranks) and was then fed to sscMap to identify compounds that could inhibit the lung tumorous phenotype, or more precisely, to identify compounds with potential to push the tumorous state closer to the normal state. Gene-signature perturbation method was employed in the subsequent connectivity mapping analysis. The details of the gene signature perturbation process, its motivation and rationale can be found in our previous work [27]. In Table 3 we show the results of connectivity mapping using this *k*=322 signature, with a perturbation stability score obtained for each drug.

**Table 3:** The significant connections returned from sscMap using the Lung cancer optimal gene signature obtained from the gene signature progression process in the case study with GSE19804 dataset

Compound	Replicates	cscore	*p* value	zscore	SM	PS
Trichostatin A	182	–0.072	1.0E-05	–4.33	1	1.00
Rofecoxib	6	–0.047	6.0E-05	–4.29	1	1.00
Calmidazolium	2	–0.110	1.0E-05	–4.23	1	1.00
MS-275	2	–0.108	7.0E-05	–3.87	1	1.00
Rifabutin	3	–0.080	7.0E-05	–3.76	1	1.00
Exemestane	1	–0.113	2.8E-04	–3.50	1	1.00
STOCK1N-35696	2	–0.082	3.0E-04	–3.38	1	1.00
1,5-isoquinolinediol	1	–0.107	4.5E-04	–3.31	1	1.00
Pioglitazone	11	–0.030	3.4E-04	–3.33	1	0.99
Gefitinib	1	–0.105	5.8E-04	–3.23	1	0.93

SM (significance mark) =1 indicates that the connection *p* value is less than the preset threshold *E*_*fp*_/*N*_*sets*_=1/1309≈7.6×10^−4^; PS (perturbation stability) is also shown here

### Breast cancer case study

As the second example of applying the gene signature progression method, we analyzed the GEO dataset GSE15852 from an independent study on human breast cancers from Malaysian patients of different ethnicity (Malays, Chinese and Indian) [28]. In total 43 pairs of tumor and normal tissues were profiled using Affymetrix HG-U133A microarrays. Similar to the lung cancer case study, the microarray experiment raw CEL files were downloaded from GEO, and processed using the MAS5 algorithm to obtain the gene expression values. Log2 transformation was applied to the MAS5 expression values, and subsequent analyses were carried out using log2Mas5 values. This dataset was based on the same microarray platform as the reference profiles in sscMap, namely the Affymetrix HG-U133A microarray platform with 22283 probestes. Paired sample t test was applied to each of the 22283 probesets individually and a two-sided *p*-value obtained for each probeset. The threshold *p*-value was set as 1/22283≈4.5×10^−5^, any probeset with a *p*-value less than this threshold was considered as statistically significant. For this case study, 1241 probesets met this criterion and were passed on to subsequent filtering. After filtering out probesets whose absolute differential expression is lower than 1, and those with mean expression levels lower than 6.0 in both conditions, finally 368 probesets were left to form the query gene signature. The filtering process described above ensured that the genes (probesets) included in the query gene signature met the criteria of both statistical significance and biological significance. These 368 probesets were finally sorted by their *p*-values in ascending order. The complete list of these probesets and their corresponding gene symbols can be found in Additional file 4.

Following the gene signature progression process we identified that *k*=232 was the minimum length for the gene signature to return significant drug connections with eFDR ≤0.1, hence this was taken as the optimal length of gene signature in our connectivity mapping analysis. Gene signature perturbation was then applied to this *k*=232 gene signature, and the perturbation stabilities calculated for each connection obtained. In Table 4 we show the results of sscMap using this *k*=232 gene signature and with gene signature perturbation procedure applied. Table 5 summarizes the key figures of the two case studies described in this paper.

**Table 4:** The significant connections returned from sscMap using the breast cancer optimal gene signature based on the case study with dataset GSE15852, comparing 43 breast tumors with their paired normal tissues

Compound	Replicates	cscore	*p* value	zscore	SM	PS
IC-86621	4	–0.079	5.0E-06	–4.84	1	1.00
Trichostatin A	182	–0.080	5.0E-06	–4.07	1	1.00
Semustine	4	–0.082	1.0E-04	–3.82	1	1.00
W-13	2	–0.091	8.0E-05	–3.77	1	1.00
Copper sulfate	4	–0.066	1.5E-04	–3.63	1	1.00
Exemestane	1	–0.136	2.8E-04	–3.58	1	1.00
Vorinostat	12	–0.087	1.9E-04	–3.47	1	1.00
Danazol	4	–0.053	2.5E-04	–3.43	1	1.00
Dexverapamil	1	–0.127	5.8E-04	–3.34	1	1.00
15-delta prostaglandin J2	15	–0.051	6.4E-04	–3.20	1	0.73

**Table 5:** Summary of the two datasets analyzed in the case studies

Dataset	GSE19804	GSE15852
Disease	Lung cancer	Breast cancer
Samples Size	120	86
Samples Details	60 tumors	43 tumors
	60 normals	43 normals
Total Genes (Probesets)	22277	22283
Threshold *P*-value	1/22277	1/22283
Significant Genes	6316	1241
Expression filtered	6066	1229
Differential expression Filtered (Fold Change > 2)	1779	368
Gene Signature Progression optimal length	322	232
Potential drugs	10	10

## Discussion

It is worth noting that there are a couple common drugs from the two case studies presented here. These are: Trichostatin A (TSA) and exemestane. Trichostatin A is primarily an antifungal antibiotic and also a potent inhibitor of histone deacetylase (HDAC) activity. Its antitumor activity against breast cancer in vitro and in vivo has already been demonstrated [29]. Several studies showed that TSA can induce apoptosis in human breast cancer cells, for instance, via targeting the mitochondrial respiratory chain and increasing mitochondrial reactive oxygen species [30], or through the involvement of 15-Lox-1 [31]. While the use of exemestane in breast cancer has been long documented [32], there is evidence that this compound also has antiproliferative effect in lung cancer cells [33], and it was even suggested as a new treatment option for mesothelioma patients [34].

Among the candidate compounds returned from the lung cancer case study, rofecoxib was shown effective inhibiting the growth of small tumors in xenograft models of non-small cell lung cancer, suggesting its potential value as adjuvant therapy after surgery [35]. Perhaps, the most interesting result is that gefitinib is among the significant drug candidates. It is interesting to note that our gene signature in the lung cancer case study was based on expression data from Taiwan female non-smoker lung cancer patients, while gefitinib has been reported in an independent study to be highly effective in treating women, non-smoker or former light smoker, advanced non-small cell lung cancer patients of Asian origin [36]. This result is indeed “spot-on” in obtaining pertinent candidate drugs.

In the two case studies presented here, we have used *p*-values to sort and rank the genes (Method M1). But this is not the only way that genes can be sorted and ranked. The other method (M2) could be employed as well. As both ranking methods (M1) and (M2) described in the “Methods: a two-stage process” section have their merits, sometimes it can be difficult to argue for one against another. The third way of ranking the genes can be a weighted combination of (M1) and (M2). For example, we can rank the genes using method (M1) and method (M2) separately, so that each gene receives two ranks, *R*_*p*_ and *R*_*e*_, based on *p* value (statistical) and on effect (biological), respectively. Then a weighted average of the two ranks can be calculated by *R*=*w*_*p*_*R*_*p*_+*w*_*e*_*R*_*e*_, where *w*_*p*_+*w*_*e*_=1. The weighted average ranks eventually determine the final ranking of genes. Finally it is also possible to incorporate external biological knowledge to adjust the ranking achieved above. Researchers will have to draw upon their expertise knowledge in the subject area and to make a decision on whether a given genes’s rank should be boosted or lowered.

## Conclusions

In this paper we introduced a gene signature progression method into connectivity mapping, which enables a standardized procedure for constructing high quality gene signatures. The essence is to determine the minimum length of the gene signature that allows significant connections returned with a target false discovery rate. This progression method is particularly useful when the number of differentially expressed genes identified is large, and when there is a need to rank and prioritize them to be included in the final query signature. On the other hand, if the number of differentially expressed genes is small, the two-stage process proposed in this paper stipulates that only the DEGs, but not others, should be included in the final gene signature. This means that, if in the end, after including all the DEGs into the query signature, the false discovery rate of returned drugs is too high, one should then be cautious in following up the returned drugs, because too high a FDR would mean a low rate of success in any experimental validation attempt. So it is important that the statistical rigor set out in the proposed two-stage process should be adhered to. The results from the two case studies demonstrate that the approach developed here is capable of obtaining pertinent candidate drugs with high precision. Future development in this area is likely to involve incorporating existing biological knowledge stored in various biological databases in a systematic and automated way, to augment the current approach based on expression data.

## Ethics approval and consent to participate

Not applicable.

## Consent for publication

Not applicable.

## Availability of data and materials

The datasets supporting the conclusions of this article are included within the article and its additional files.

## Additional files

Additional file 1 Significant genes associated with HDAC inhibitors. The full list of 104 genes (probesets) and their rank order, obtained in the “simulated experiment” with 4 HDAC inhibitors of the CMap reference profiles. (TAB 6.85 kb)

Additional file 2 Significant drugs returned with the *k*=50 HDACi gene signature. The list of significant drugs returned from sscMap using the *k*=50 HDACi gene signature from the “simulated experiment”. All 7 known HDAC inhibitors were pulled out, but together with many other drugs, which may or may not related to HDAC inhibition. The main biological theme (HDAC inhibition) could potentially be weakned or even masked due to the co-existence of many other drugs. (XLSX 17 kb)

Additional file 3 Lung cancer gene signature. The full list of 322 genes (probesets) and their ranks for the optimal gene signature obtained from the lung cancer dataset GSE19804. (TAB 41.1 kb)

Additional file 4 Breast cancer gene signature. The full list of 232 genes (probesets) and their ranks for the optimal gene signature obtained from the breast cancer dataset GSE15852. (TAB 29.9 kb)

## Abbreviations

CMap: connectivity map
DEG: differentially expressed gene
eFDR: empirical false discovery rate
FDR: false discovery rate
GEO: gene expression Omnibus
HDAC: histone deacetylase
HDACi: histone deacetylase inhibitor
TSA: trichostatin A
